# Trio, a Rho Family GEF, Interacts with the Presynaptic Active Zone Proteins Piccolo and Bassoon

**DOI:** 10.1371/journal.pone.0167535

**Published:** 2016-12-01

**Authors:** Ryan T. Terry-Lorenzo, Viviana I. Torres, Dhananjay Wagh, Jose Galaz, Selene K. Swanson, Laurence Florens, Michael P. Washburn, Clarissa L. Waites, Eckart D. Gundelfinger, Richard J. Reimer, Craig C. Garner

**Affiliations:** 1 Dept. of Psychiatry and Behavioral Science, Nancy Pritzker Laboratory, Stanford University, Palo Alto, California, United States of America; 2 Centro de Envejecimiento y Regeneración (CARE), Departamento de Biología Celular y Molecular, Facultad de Ciencias Biológicas, Pontificia Universidad Católica de Chile, Santiago, Chile, Alameda, Santiago, Chile; 3 Stowers Institute for Medical Research, Kansas City, Missouri, United States of America; 4 Dept. of Neurochemistry and Molecular Biology, Leibniz Institute for Neurobiology, Magdeburg, Germany; 5 Dept. of Neurology and Neurological Sciences Stanford University and Veterans Affairs Palo Alto Health Care System, Palo Alto, California, United States of America; 6 German Centers for Neurodegenerative Diseases, Charité - Medical University, Berlin, Germany; Karolinska Institutet, SWEDEN

## Abstract

Synaptic vesicles (SVs) fuse with the plasma membrane at a precise location called the presynaptic active zone (AZ). This fusion is coordinated by proteins embedded within a cytoskeletal matrix assembled at the AZ (CAZ). In the present study, we have identified a novel binding partner for the CAZ proteins Piccolo and Bassoon. This interacting protein, Trio, is a member of the Dbl family of guanine nucleotide exchange factors (GEFs) known to regulate the dynamic assembly of actin and growth factor dependent axon guidance and synaptic growth. Trio was found to interact with the C-terminal PBH 9/10 domains of Piccolo and Bassoon via its own N-terminal Spectrin repeats, a domain that is also critical for its localization to the CAZ. Moreover, our data suggest that regions within the C-terminus of Trio negatively regulate its interactions with Piccolo/Bassoon. These findings provide a mechanism for the presynaptic targeting of Trio and support a model in which Piccolo and Bassoon play a role in regulating neurotransmission through interactions with proteins, including Trio, that modulate the dynamic assembly of F-actin during cycles of synaptic vesicle exo- and endocytosis.

## Introduction

Active zones (AZ) are specialized regions of the presynaptic plasma membrane of neurons designed to regulate the activity-dependent release of neurotransmitter [1]. AZ proteins are thought to function in concert with trans-synaptic cell-adhesion molecules (CAMs) to define sites of neurotransmitter release, holding them in register with the postsynaptic density (PSD) [1, 2]. In addition, they are thought to precisely coordinate aspects of the synaptic vesicle (SV) cycle [3] such as the translocation of SVs from the reserve to the readily releasable pool, and SV exocytosis/endocytosis [1].

Ultrastructural studies of excitatory and inhibitory synapses suggest that, similar to other membrane specializations [4], AZs are delineated by a collection of scaffold/cytoskeletal proteins [2]. The molecular analysis of the CAZs has lead to the identification of a number of large multi-domain proteins [1]. One group, including Munc13 and RIM1α have been linked to the priming of SVs during exocytosis at the AZ as well as short and long-term forms of presynaptic plasticity [5]. A second group, including ELKS (also referred to as ERCs or CAST), Liprin, Bassoon, and Piccolo, have been implicated in the structural assembly of the AZ, creating a lattice that guides SVs to neurotransmitter release sites [2]. For example, the loss of Liprin/SYD2 in *C*. *elegans* leads to disorganized or absent AZs [6–8]. Similarly, in photoreceptor cell ribbon synapses lacking Bassoon, the ribbons, specialized structures of the CAZ, are detached from the presynaptic membrane, consequently impairing vision [9].

It remains unclear whether proteins of the CAZ are predominantly engaged in scaffolding other AZ proteins or, as in the case of RIM1α directly participate in the priming and recycling of SVs [10, 11]. In the case of Piccolo and Bassoon, their large size, multi-domain nature, and early arrival at nascent synapses suggests they play a fundamental role in the assembly of the AZ [2]. Although the individual loss of Piccolo [12, 13] or Bassoon [14] from synapses does not cause major defects of AZ assembly, the simultaneous loss of both these proteins from hippocampal neurons in culture induces ubiquitination and degradation of presynaptic proteins, suggesting a shared role for both proteins in synaptic integrity [15]. To date, two classes of molecules have been found to interact with Piccolo. The first class includes other CAZ proteins such as ELKS [16], Liprins [17], RIM1/2 [18], voltage gated calcium channels (VGCC) [18] and/or Ribeye [19]. The second class includes, regulators of the actin cytoskeleton such as Profilin1/2 [20], Abp1 [21], GIT1 [22], and Daam1 [23].

Importantly, the efficiency and plasticity of SV exocytosis and endocytosis depends on the dynamic assembly and disassembly of F-actin [24–27]. F-actin interacts with Synapsin to regulate the translocation of SV from the reserve pool (RP) to the readily releasable pool (RRP) [24] and through its interaction with Dynamin, Abp1, and Synapsin regulates SV endocytosis [25, 28, 29]. Interestingly, we have unraveled a role for Piccolo in SV traffic from the RP to the RRP. In this role, Piccolo modulates Synapsin1a dynamics [12] and presynaptic F-actin assembly [30], the latter involving Profilin1, CaMKII [30, 31], and Daam1 [23].

Roles for Bassoon described so far involve synaptic plasticity through its interaction with the adaptor protein 14-3-3 [32] and recruitment of P/Q-type calcium channels close to release sites through RIM-binding protein [33]. At vertebrate sensory synapses, it has been shown that Bassoon is key in the anchoring of ribbons to the AZ of photoreceptor [9] and inner hair cells [34]. However, unlike Piccolo, there is no report implicating Bassoon in AZ F-actin assembly. Therefore, Piccolo and Bassoon, through their multi-domain structure, have unique and shared functions regulating molecular AZ processes [2].

To gain further clues into how Piccolo and Bassoon regulate presynaptic function, we performed a biochemical/proteomic analysis of proteins found in complexes with Piccolo and Bassoon in immature brains, at a time when many CAZ proteins are being transported to nascent synapses in association with transport vesicles [35–38]. This approach led to the identification of Trio, a member of the Dbl family of Rho-guanine nucleotide exchange factors (Rho-GEF), as a novel Piccolo/Bassoon binding partner. Intriguingly, Trio has previously been found to regulate the assembly of the actin cytoskeleton during axon guidance, neurite outgrowth, and the secretion of peptides from endocrine cells [39, 40]. Our present study reveals that Trio is targeted to presynaptic boutons via its association with Piccolo and Bassoon, where it is situated to modulate the dynamic assembly of F-actin.

## Materials and Methods

### Primary antibodies

Antibodies against (α-) Piccolo (rabbit), Bassoon (mouse), and MAP2 (rabbit and mouse) were used as previously described [41]. α-Tubulin (mouse) antibodies were from Sigma, and α-PSD-95 (mouse) was from Affinity BioReagents. The following antibodies were purchased from Santa Cruz: α-Synaptophysin (rabbit), α-Trio (C-terminal antibody; goat), and α-Myc (rabbit). The mouse Trio α-GEF2 antibody was from Abnova. Mouse α-Synaptotagmin was purchased from BD Biosciences. The rabbit α-GFP antibody was from Invitrogen. The α-ELKS2 antibody was generated in rabbits using a commercial vendor (Washington Biotechnology). The epitope was *E*. *coli* purified GST-tagged amino acids 107–138 of ELKS2 [same region as used by [16]]. The serum was passed over a column of GST coupled to Actigel ALD using manufacturer’s protocol (Sterogene Bioseparations) to remove antibodies directed against GST. Antibody was then affinity purified with the antigen coupled to Actigel ALD.

### DNA plasmid construction

The human Trio cDNA (accession number AF091395) in EGFP-C1 vector (Clontech) was provided by Dr. Anne Debant [42]. Myc-tagged Trio was generated by excising the GFP cassette with AgeI and BspEI and replacing it with a synthetic oligonucleotide encoding the Myc epitope. C-terminal truncated Trios were generated by using blunt end restriction endonucleases and ligating the resultant plasmid using the SmaI site in the EGFP backbone. These molecular biology reagents were purchased from New England Biolabs. Myc-tagged polypeptides encompassing various Spectrin repeats of Trio were generated by standard PCR cloning methodologies. The cDNA of full length (FL) Myc-Trio lacking amino acids 395–606 (Spectrin repeats #3 and #4) was generated using the gap overlap extension method of PCR. All PCR cloning was conducted with KOD DNA Polymerase (Novagen) and resultant cDNA products were sequenced to ensure fidelity.

The GFP-tagged rat Bassoon cDNA was a kind gift from Thomas Dresbach and Wilko Altrock [43]. To generate the cDNA encoding amino acids 4077–4875 of rat Piccolo (accession number NM_020098), we excised C-terminal PBH9/10-PDZ-C2A region Piccolo from the R2 plasmid [44] with EcoRI and ligated this insert into the EcoR1 site in EGFP-C2 (Clontech).

### Identification of Trio

To identify novel proteins associating with Piccolo and Bassoon in young brains, we utilized two approaches. For the first approach, we used a three-step protocol that separates lipid-associated proteins, such as Piccolo and Bassoon, based upon their buoyant density, size, and charge (S1A Fig). All steps were conducted either on ice or at 4°C, using P4 Sprague Dawley rats (Charles River) that were euthanized with carbon dioxide and decapitated. Step (1) involved a sucrose density gradient centrifugation in which ~100 postnatal day 4 rat brains were homogenized in buffer A (5 mM MES, pH 7.0, 0.3 M sucrose, 1 mM EDTA) containing protease inhibitor cocktail (Roche Biochemicals) with 10 strokes in a Teflon-glass homogenizer. The homogenate was centrifuged at 1,000xg for 15 min. Post-nuclear supernatant (PNS) was hypotonically lysed with 10 volumes of 5 mM MES pH 7.0, 1 mM EDTA, and incubated for 30 min at 4°C. The lysed PNS was then centrifuged at 100,000xg for 1 h. The pellet (P100) was resuspended in buffer A and loaded on top of a discontinuous sucrose gradient of 0.8, 1.2, and 2.0 M. The gradient was spun for 3 h at 270,000xg in a SW28 or SW 41Ti. The material between 0.3 and 0.8 M sucrose was removed (~40 mg), diluted with 5 mM MES, pH 7.0, 1 mM EDTA, to a final sucrose concentration of 0.3 M, and centrifuged at 100,000 x g for 1 hour. The pellet was further resuspended in a final volume of 2 mL of PBS with protease inhibitors. In step (2), this material was applied to a gel filtration column packed with Sephacryl S-1000 Superfine matrix (Amersham Biosciences). The dimensions of the column were 1.5 cm wide and approximately 1.5 meters tall. Material flowed through the column by gravity with elution by 1x phosphate buffered saline (PBS, pH 7.4). The flow rate was ~0.5 mL/minute, and a BioRad Model 2110 fraction collector was used to collect 80 fractions of 4 mL per fraction. For the final step (3) of the protocol, the four Sephacryl S-1000 fractions most highly enriched in Piccolo and Bassoon as determined by Western blotting were pooled (at this point about 1 mg in 15 mL) and applied to a column packed with Q Sepharose Fast Flow (Amersham Biosciences). The dimensions of this column were diameter of 2.5 cm, height of approximately 12 cm with a bed volume of 60 mL packed Q Sepharose, and the flow rate using the 2110 fraction collector was 3 mL/minute and 10 mL fractions were collected. After optimization to separate Bassoon- and Piccolo-enriched from Synaptophysin-enriched samples, the final elution profile (all buffers in 20 mM Tris pH 7.0) was 200 mL of 150 mM NaCl to wash, then stepwise elution of 120 mL buffer with the following NaCl concentrations: 210 mM, 290 mM, 350 mM, and 1000 mM. Approximately 40 mL of the 210 mM, 290 mM (Piccolo and Bassoon enriched), and 350 mM NaCl fractions were concentrated to about 1 mL using Amicon Centricon Plus-20 30 kDa cutoff concentrators (Millipore). The final recovery was 7.5 μg in the 210 mM fraction, and 16 μg in each of the 290 mM and 350 mM fractions. The fractions were precipitated with tricholoroacetic acid, and the protein pellet was stored at -20°C until further use.

The second approach used to identify proteins associating with Piccolo and Bassoon was an anti-Piccolo IP. Here the starting material was 10 mg of the fraction between 0.3 and 0.8 M sucrose after density centrifugation (step #1 above). This material was diluted to 0.3 M sucrose with 5 mM MES, pH 7.0 and centrifuged at 100,000 x g for 1 hour. The pellet was resuspended and incubated for 1 hour with 4 mL of solubilization buffer (5 mM MES, pH 7.0, 150 mM NaCl, 1 mM EDTA, 1% Triton X-100, and protease inhibitor cocktail). Lysate was cleared by centrifugation at 100,000xg for 1 hour. One mL of supernatant was incubated with 2.5 μg α-Piccolo rabbit antibody or 2.5 μg rabbit IgG for 4 hours. A 100 μL Protein A agarose slurry (Roche) was then added and further incubated overnight. Material was transferred to a small column and washed with 20 mL solubilization buffer. Proteins were eluted twice with 200 μL 50 mM Tris, pH 6.5, 100 mM DTT, 2% SDS. This material was then extracted with 4 volumes methanol and 1-volume chloroform. This mixture was combined with water and centrifuged for 90 seconds at 16,000xg. The aqueous phase was removed and extracted again with 3 volumes of methanol. This precipitated protein was collected by centrifugation for 90 seconds at 16,000xg, methanol removed, and the speed-vacuum was used to dry the protein pellet.

The 210 mM, 290 mM, and 350 mM NaCl fractions from the Mono-Q column protocol and the eluates (both IgG alone and α-Piccolo IPs) from two separate IP experiments were subjected to Multi-dimensional Protein Identification technology (MudPIT). TCA-precipitated proteins were urea-denatured, reduced, and alkylated, then digested with endoproteinase Lys-C (Roche) followed by trypsin (Roche) as described previously [45]. The peptide mixture was loaded onto a 100 μm fused silica microcapillary column packed with 5-μm C18 reverse phase material (Aqua, Phenomenex), followed by 5-μm Strong Cation Exchange resin (Partisphere SCX, Whatman), and reverse phase. Loaded microcapillary columns were placed in-line with a Quaternary Agilent 1100 series HPLC pump coupled to a Deca-XP ion trap mass spectrometer equipped with a nano-LC electrospray ionization source (ThermoScientific). Fully automated 6-step chromatography runs were carried out on the electrosprayed peptides [46]. Tandem mass (MS/MS) spectra were interpreted using SEQUEST against a database of 86132 sequences, consisting of 42882 non-redundant rat proteins (downloaded from NCBI on 2014-08-09), and 193 usual contaminants (human keratins, IgGs, and proteolytic enzymes), and, to estimate false discovery rates, 43066 randomized amino acid sequences derived from each non-redundant protein entry. Peptide/spectrum matches were sorted and selected using DTASelect [47] with the following criteria set: spectra/peptide matches were only retained if they had a DeltCn of at least 0.08 and, minimum XCorr of 1.8 for singly-, 2.0 for doubly-, and 3.0 for triply-charged spectra; in addition, the peptides had to be fully-tryptic and at least 7 amino acids long. Combining all runs, proteins had to be detected by at least 2 such peptides, or 1 peptide with 2 independent spectra. Peptide hits from multiple runs were compared using CONTRAST [47]. To estimate relative protein levels, distributed Normalized Spectral Abundance Factors (dNSAFs) were calculated for each detected protein, as described in [48].

The complete MudPIT mass spectrometry dataset (raw files, peak files, search files, as well as DTASelect result files) can be obtained from the MassIVE database via ftp://MSV000079897@massive.ucsd.edu/ with a password RTL40349. The ProteomeXchange accession number for this dataset is PXD004552.

Positives from the Mono-Q column were defined as proteins that were absent from the 210 mM fraction and had at least 5 fold more peptides identified in the 290 mM (Piccolo/Bassoon enriched; S2 and S3 Figs) vs. the 350 mM NaCl fractions. Positives from the IP approach were defined by presence in the α-Piccolo, but not IgG alone IP fraction.

### Cell culture and transfection

Hippocampal cultures were prepared using a modified Banker culture protocol [49]. Briefly, time pregnant Sprague Dawley rats (Charles River) were euthanized with carbon dioxide and decapitated and embryos (E18) harvested. Hippocampi from embryos were removed and dissociated in 0.05% trypsin (Invitrogen), and neurons plated at a density of 165/mm^2^ on poly-L-lysine coated coverslips (Carolina Biological). One hour after plating, coverslips were transferred in pairs to 60 mm dishes containing a glial feeder layer, where they were inverted (to maximize neuronal contact with secreted glial factors) and maintained in Neurobasal medium (NB) containing B27 and GlutaMAX (all from Invitrogen).

Transfections in COS7 cells were conducted with Lipofectamine 2000 using manufacturer’s protocols (Invitrogen). For a 3.5 cm dish, 2 μg total DNA and 5 μL lipofectamine 2000 were used for the transfection, and other sized dishes scaled up using a similar DNA:lipofectamine ratio. Neuronal transfections were conducted using either lipofectamine 2000 or a calcium phosphate precipitation procedure. For the lipofectamine 2000 protocol, 2.5 μg DNA was mixed with 62.5 μL plain NB, and separately, 2.5 μL lipofectamine 2000 was mixed with 62.5 μL plain NB. After a 5 minute incubation, these tubes were mixed and incubated for 20 minutes. Neurons were placed face up in 3.5 cm dishes with 1 mL glial-conditioned media. DNA/lipofectamine complexes were added. After 30–60 minutes, the neurons were returned to their glial feeder layer. Cultured neurons also were transfected by calcium phosphate precipitation. Briefly, 2 μg DNA and 7.5 μL 2M CaCl_2_ in 60 μL total volume was added dropwise to 60 μL 2 x HBS (274 mM NaCl, 10 mM KCl, 1.4 mM Na_2_HPO_4_, 15 mM glucose, 42 mM HEPES, pH 7.1) and precipitated for 20 min in the dark. The DNA/Ca_3_(PO_4_)_2_ precipitate was then pipetted onto a coverslip of cultured neurons in 1 ml conditioned media containing 10 μM CNQX and 50 μM APV. Neurons were incubated at 37°C / 5% CO_2_ for 30 min, rinsed three times with 2 ml pre-warmed HBSS and transferred back into culture dishes.

### Protein biochemistry protocols

To generate post-nuclear supernatants (PNS), whole rat brains were homogenized in 5 mM MES pH 7.0, 320 mM sucrose, 1 mM EDTA, with an amount of protease inhibitor cocktail tablet as directed by the manufacturer (Roche). Homogenates were centrifuged at 1000xg for 20 minutes; supernatant was then removed and stored. Postsynaptic densities (PSD) from adult rat forebrains were generated using a standard protocol [50]. Of note, the final solubilization of synaptosomes was conducted for 15 minutes with a buffer containing 1% Triton X-100.

IPs from COS7 cells were conducted in the following manner: 6 cm dishes of transfected, confluent COS7 cells were washed 1x in phosphate-buffered saline. One mL of lysis buffer (50 mM Tris, pH 7.5, 150 mM NaCl, 1 mM EDTA, 1% Triton X-100, protease inhibitor tablet) was added, cells were scraped off and placed into a tube for a one-hr incubation. Lysate was cleared at 20,000xg for 20 minutes. 450 μL lysate was mixed in a tube with 1 μg either rabbit α-Myc Ab (Santa Cruz) or rabbit IgG (Upstate) and incubated for 1 h. 40 μL protein A/G bead slurry (Pierce) was added and mixture was incubated for 1 hour. Beads were centrifuged at 500xg and washed in NETN-250 (20 mM Tris, pH 7.5, 1 mM EDTA, 250 mM NaCl, 0.5% NP-40) 4 times. Protein was eluted and prepared for SDS-PAGE by incubation with 40 μL of sample buffer (62.5 mM Tris, pH 6.8, 10% glycerol, 2% SDS, 5% β-mercaptoethanol, and Bromophenol Blue).

For Western blotting, protein samples were separated by SDS-PAGE using 3–8% gradient Tris-acetate gels (Invitrogen), transferred to nitrocellulose membranes (Amersham), incubated in blocking solution (5% Nonfat dry milk, 0.05% NP-40, 150 mM NaCl, 50mM Tris, pH 7.5), and probed with primary and secondary horseradish peroxidase (HRP)-conjugated antibodies (GE Healthcare) in blocking solution. Protein bands were visualized by HRP chemiluminescence (PerkinElmer) and exposure to film. For Western blot quantification, the developed film was scanned, images stored as (.tiff) files, and Image J software was used to measure band intensity with appropriate background subtraction.

### Immunohistochemistry and imaging

Hippocampal neuronal cultures or COS7 cells were fixed with either 4% formaldehyde in PBS for 10 min, or 100% ice-cold methanol for 20 min. Cells were then permeabilized with 0.25% Triton X-100 in PBS for 5 min, washed in PBS, incubated in blocking solution (2% bovine serum albumin, 2% glycine, and 0.2% gelatin in 50mM NH_4_Cl) for 30 min at room temperature, and incubated overnight at 4°C with primary antibodies in blocking solution. Afterwards, cells were rinsed 3–4 times in PBS, incubated for 1 hr at room temperature with secondary antibodies in blocking solution, rinsed again 3–4 times in PBS followed by a final rinse in deionized water, dried, and mounted in Vectashield mounting solution (Vector Laboratories, Inc.). Primary antibodies were detected either with goat α-mouse or α-rabbit antibodies conjugated with Alexa 488, 568, or 647 (Invitrogen/Molecular Probes). When imaging endogenous Trio with the goat α-Trio antibody, we used only methanol fixation, and we used donkey α-mouse Cy5 and α-rabbit Cy2 (Jackson) for co-staining other proteins. Of note, the Alexa647 and Cy5 “far red” panels are presented as blue in merged images throughout the manuscript.

Neurons and COS7 cells were imaged with a Zeiss Axovert 200M microscope using a 63x objective. The image emission was directed through a CSU10 spinning disk confocal unit (Yokogawa) and collected by a 512B-CCD camera (Roper Scientific). All imaging acquisition and analysis was conducted with Metamorph software. With a Z step size of 1 μm, stacks of images were collected and Metamorph tools were used to create a maximum projection image. Both qualitative and quantitative analyses were conducted by a scientist blind to cell identity. All experiments were repeated greater than 3 times.

For the COS7 clustering assay, Myc-tagged Trio proteins were scored as either positive (+) or negative (-) for clustering with the punctal GFP-Bassoon. Penetrance for the positive clustering was greater than 70%, and negative clustering was defined as fewer than 10% of cells exhibiting co-clustering of Myc-tagged with GFP protein; only in one condition was an intermediate phenotype (denoted as “-/+”) observed.

The Metamorph cell scoring function was used for quantification of protein co-localization. Briefly, all images were thresholded. Then, the PSD-95 image was used to create a mask to generate PSD-95 positive and negative regions of the image. The PSD-95 masks were applied to Piccolo or Synaptophysin thresholded images to separately pool PSD-95 positive and negative puncta of Piccolo or Synaptophysin. Finally, these puncta were scored as either Trio positive or negative. Experiment was repeated 3 times with essentially similar results, and the reported results were based upon an experiment with an N (defined as a single image) of 10.

### Ethics statement

All animal studies were approved by the Stanford University Animal Care and Use Committee.

## Results

### Identification of Trio as a member of Bassoon/Piccolo complexes in immature rat brain

The purification of active zone-specific protein complexes has been hampered by the inability to biochemically separate the CAZ from its corresponding postsynaptic compartment, the postsynaptic density (PSD) [51]. Thus, to identify in vivo protein complexes formed by Piccolo and Bassoon, we developed a biochemical strategy that took advantage of the fact that these proteins are transported to synapses on vesicles with other AZ proteins, such as ELKS but not with SV or postsynaptic proteins in the developing brain [35, 38, 52]. To identify proteins that form complexes with Piccolo and other tightly bound CAZ proteins, we conducted an immunoprecipitation (IP) of Piccolo from the sucrose gradient centrifugation light membrane fraction of postnuclear supernatants (PNS) from immature (2–4 day old) rat brains. Material from these IPs were initially tested by Western blotting to confirm the presence of known interacting proteins (Bassoon, ELKS2, and RIM1/2; S3B Fig) and then subjected to Multi-dimensional Protein Identification Technology (MudPIT), a sensitive proteomic technique capable of identifying proteins in complex cellular mixtures [46, 48]. The MudPIT analysis identified a total of 24 proteins that were identified in the Piccolo IP, but absent in the control IgG (See S1 Table).

To increase our ability to eliminate false positives identified in IP, we used an orthogonal approach to identify proteins that interact with Piccolo. Specifically, we subjected light membrane fraction from immature rat brains to size exclusion chromatography, and anion exchange chromatography to separate macromolecule complexes based upon the properties of density, size, and charge, respectively (S1A Fig). After some degree of optimization, it was possible to isolate several fractions from the final Q Sepharose column (S1B, S2 and S3A Figs) with distinct protein compositions. Of particular interest was the 290 mM NaCl elution fraction, which was highly enriched in Piccolo and Bassoon, contained ELKS2, and was devoid of the synaptic vesicle (SV) protein synaptophysin (Syph). The 210 nM, 290 mM, and 350 mM NaCl elution fractions were subjected to MudPIT, with the goal of identifying proteins similarly enriched in the 290 mM fraction.

While a great benefit of the MudPIT technique is its sensitivity and capacity to identify multiple proteins in complex mixtures, a challenge is separating true positives from false positives. In particular, high abundance contaminants are often found, confounding assessment of which identified proteins are legitimate interactors with target proteins of interest. Indeed, our workflow identified a great number of proteins in our biochemical fractions (see S1 Table). The proteins are quantified by the distributed spectral counts (dSpC) metric, which is the number of tandem mass spectra that match peptides from a particular protein; this value is then converted into a distributed normalized spectral abundance factor (dNSAF) value [48] to assist in semi-quantitative comparisons among proteins.

To assess likelihood of a protein being a legitimate member of Piccolo/Bassoon protein complexes, we prioritized proteins (1) detected in the 290 mM, but not 210 mM and 350 mM fractions, and (2) detected in the Piccolo co-IP, but not the control IgG IP. Our defining CAZ proteins, Piccolo and Bassoon, fulfilled these criteria fully (Table 1). In contrast, >30 spectra of ELKS isoforms were identified in both the 210 mM and 290 mM fractions, with no peptides identified in the 350 mM fraction; this result is fully consistent with our identification of ELKS2 in these fractions by Western blots (S2 Fig). Other known AZ proteins, such as Liprin-α, Munc-18, and CASK, were identified in the Piccolo/Bassoon-enriched 290 mM fraction, but with the exception of CASK, peptides of these proteins were also found in other fractions as well. While the fractionation protocol generally identified more proteins that the co-immunoprecipitation (IP) protocol (S1 Table), one exception was the known Piccolo-interacting protein Daam1 (38), where 3 peptide spectra were identified in the Piccolo co-IP (Table 1).

**Table 1:** Proteins co-enriched with Piccolo. Two protocols to biochemically enrich for Piccolo and other CAZ proteins (column chromatography & immuno-precipitation) were subjected to MudPIT to identify co-purifying proteins. The most interesting proteins would fulfill two criteria: (1) They would be detected in the 290 mM NaCl fraction, but not in the 210 mM or 350 mM fractions; (2) They would be detected in the Piccolo antibody (Ab) co-IP, but not in the control IgG IP. Piccolo, Bassoon, and Trio were the only three proteins that fulfilled these criteria. Also shown in this table for comparison are the peptides identified for several CAZ proteins and Piccolo/Bassoon interacting proteins. For each protein in each biochemical fraction are shown the number of Distributed Spectral Counts (dSpC) identified, along with the Distributed Normalized Spectral Abundance Factor (dNSAF)[48].

	Fractionation Protocol; Q column eluates						Immunoprecipitation Protocol							
	210 mM		290 mM		350 mM		Piccolo Ab, IP#1		Piccolo Ab, IP#2		Control IgG, IP#1		Control IgG, IP#2	
Protein Name	dSpC	dNSAF	dSpC	dNSAF	dSpC	dNSAF	dSpC	dNSAF	dSpC	dNSAF	dSpC	dNSAF	dSpC	dNSAF
Piccolo			12	0.000625			159	0.025937	10	0.004332				
Bassoon			1	0.000067			2	0.000422	1	0.000560				
CAST2/ELKS	31	0.008277	35	0.008619										
Liprin α2	1	0.000202	2	0.000424	1	0.000117								
CASK			2	0.000486										
Git1			24	0.008130										
Git2			3	0.001178										
Munc-18	9	0.003778	3	0.001318							1	0.001223		
Daam1	1	0.000262							3	0.006826				
Trio			7	0.000598			2	0.000535						

munc-18 = syntaxin binding protein 1

In the entire list of proteins identified, our attention was directed to the one protein other than Piccolo and Bassoon that was both detected with multiple peptides specifically in the 290 mM fraction (not found in the 210 mM and 350 mM fractions) and identified by Piccolo co-IP. That protein was the Rho-GEF Trio (Table 1). A Western blot of proteins enriched in the 290 mM NaCl fraction from the Q Sepharose column was used to verify the presence of Trio, like Piccolo and Bassoon, in this fraction (S3A Fig).

### Developmental expression of Trio, Piccolo and Bassoon

**Fig 1:**
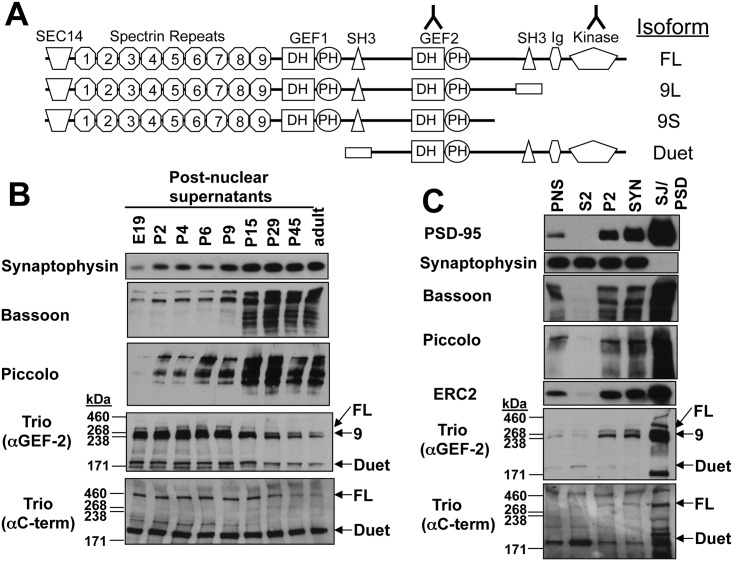
Developmental expression of Trio isoforms and their association with synaptic junctions. (A) Schematic diagram depicting rat forebrain isoforms of Trio as previously defined [62]. Inverted “Y”s depict the epitopes recognized by the two antibodies (αGEF-2 and αC-term) used throughout this study. (B) Western blots of postnuclear supernatants (PNS) generated from total rat brains at embryonic (E) or postnatal (P) ages. Note that while SV (Synaptophysin) and CAZ (Bassoon and Piccolo) proteins were upregulated during development, Trio isoforms (denoted by arrows to the right of blots) were partially downregulated. (C) Western blots depicting the amounts of PSD-95, Synaptophysin, Bassoon, Piccolo, ELKS2, and Trio in the following fractions successively prepared from adult rat forebrain: PNS, supernatant/cytosolic (S2), pellet (P2), synaptosomal (SYN), and synaptic junction/postsynaptic density (SJ/PSD). Although the smallest Trio isoform (Duet) was enriched in the S2 fraction, the three larger isoforms were enriched in the SJ fraction, similar to PSD-95, Bassoon, Piccolo, and ELKS2. Note, while the SJ is traditionally referred to as the PSD fraction, this is a misnomer as it contains scaffold proteins of both the PSD and AZ.

Trio is a large multidomain protein conserved throughout evolution [53]. Its N-terminal region contains a SEC14 lipid interacting domain and nine Spectrin-like repeats, which are known to mediate protein-protein interactions (Fig 1A). The C-terminal portion of mammalian Trio contains the 3 enzymatic domains (two functional guanine nucleotide exchange factor (GEF) domains and a protein serine/threonine kinase (PSK) domain) that define its name, as well as additional protein interaction domains, including an Ig domain and two SH3 domains. Among its enzymatic activities, studies in *C*. *elegans*, *Drosophila*, and mammalian systems have demonstrated that the GEF regions appear to be particularly important for the biological functions of Trio [54–58]. Trio is a member of the large Dbl homology (DH)/ pleckstrin homology (PH) domain proteins known to activate Rho family GTPases via GEF activity [59]. Rho family GTPases, in turn, regulate actin-based cellular morphology changes in a variety of cell types [59–61] as well as SV docking and fusion [61].

Mammalian Trio exists as several isoforms [62, 63] (Fig 1A). The 9 Trio peptides identified by MudPIT (Table 1) were from protein regions common to multiple Trio isoforms, such that the MudPIT results did not provide evidence that any Trio isoforms selectively co-enriched with Piccolo. To begin to assess which Trio isoform(s) may be relevant to Piccolo/Bassoon association, we employed two commercial antibodies: one directed against the C-terminus that can identify the Full length (FL) and Duet isoforms of Trio, and a second one directed against the GEF-2 region that can identify all 4 isoforms of Trio found in the rat forebrain, including the 9L and 9S isoforms ([62]; Fig 1A). Of note, these two antibodies are unable to identify an additional isoform of Trio named Trio8/Solo [62, 64] that has restricted expression in the cerebellum. In Western blots of post-nuclear supernatant fractions, FL-Trio was recognized by both antibodies as a band between the 268 and 460 kDa molecular weight markers; the 9L and 9S isoforms were clearly delineated as a doublet around 240–270 kDa (only recognized by the α-GEF2 antibody); and the Duet isoform was seen as a band slightly above the 171 kDa molecular weight markers (Fig 1B and 1C). This latter band was a slightly higher molecular weight than previously reported [62], but is likely the Trio Duet isoform, as it was recognized distinctly by both antibodies, and was the major band in crude brain lysates recognized by the C-terminal antibody (Fig 1B and post nuclear supernatant (PNS) lane in Fig 1C).

To examine the developmental distribution of Trio relative to Piccolo and Bassoon, postnuclear supernatants (PNS) from equal mass quantities of brain from rats of 9 different ages were analyzed by western blot. Synaptic proteins, including Synaptophysin, Bassoon, and Piccolo, were up-regulated during brain development (Fig 1B) as synapse density increases. Conversely, the four Trio isoforms detected by the two antibodies were expressed at high levels at the earliest time point explored (embryonic day 19), and with the exception of Duet, were down-regulated as the brain matured past postnatal day 15 (P15)(Fig 1B). Thus, while Trio, Piccolo, and Bassoon were co-expressed in the brain at multiple developmental time points, their divergent developmental regulation suggests they may have functions both dependent and independent of their putative association.

### Trio co-localizes with Piccolo/Bassoon at synapses

We sought to determine if Trio, Piccolo, and Bassoon have similar subcellular localizations. Piccolo and Bassoon exhibit a varied subcellular localization depending on the stage of brain development. For example, during early brain development, these CAZ proteins are found in association with Piccolo/Bassoon Transport Vesicles (PTVs) [35], the Golgi apparatus [38, 65], growth cones [41, 66], and nascent synapses [35, 67]. In mature brains, Piccolo and Bassoon are predominately tightly associated with the CAZ [66, 68] though low levels are found in association with Golgi membranes and PTVs. The subcellular neuronal localization of Trio is less well-understood [69].

We therefore performed a series of experiments to compare the subcellular distribution of Trio, Piccolo, and Bassoon, with a primary focus on their potential colocalization at synapses. Initially, we assessed the ability of Trio isoforms to be enriched in purified SJ/PSD preparations known to also contain CAZ proteins [51, 70]. Western blots of 5 fractions generated during the purification of synaptic junctions from adult rat brain revealed the expected enrichment of PSD-95 in the SJ/PSD fraction (Fig 1C). In contrast, the synaptic vesicle (SV) protein synaptophysin was enriched in the synaptosomal (SYN) fraction, but not the Triton-extracted SJ/PSD fraction (Fig 1C). Similar to PSD-95, the CAZ proteins Piccolo, Bassoon, and ELKS2 were enriched in the synaptic junctional preparations (Fig 1C). Intriguingly, the FL, 9L, and 9S isoforms of Trio were also highly enriched in these preparations (Fig 1C). These results suggest that, while most Trio isoforms were down regulated during brain development (Fig 1B), the high molecular weight Trio isoforms (FL, 9S, 9L) that continue to be expressed in the adult brain were highly enriched in synaptic junctions (Fig 1C). Intriguingly, the Duet isoform was more abundant in the S2 cytosolic fraction and mostly absent from the SJ/PSD fractions (Fig 1C). These data suggest that the N-terminal regions of Trio (see Fig 1A) may be required for synaptic targeting.

**Fig 2:**
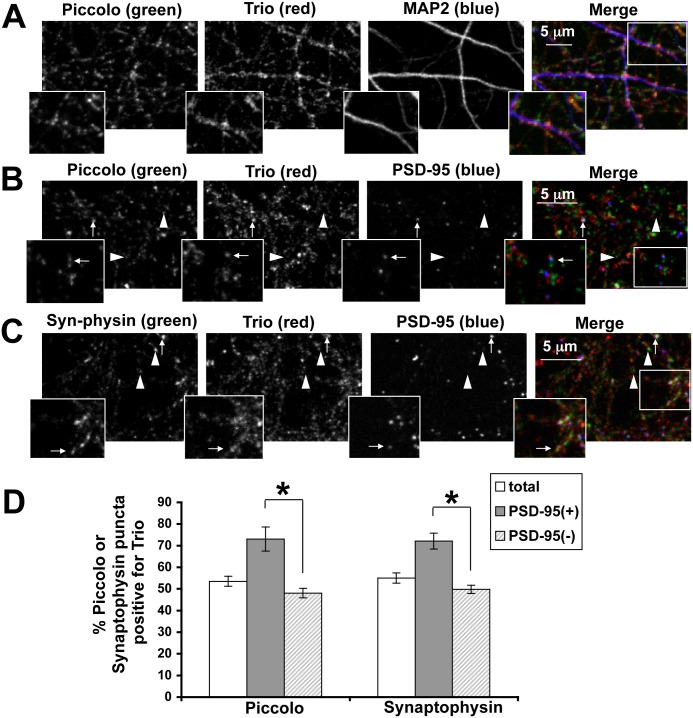
Trio localizes to synapses in cultured hippocampal neurons. (A, B, and C) Images of 16–19 DIV neurons fixed in methanol and immunostained for the proteins indicated. Arrows depict puncta of either Piccolo or Synaptophysin that are positive for PSD-95 and, thus, likely excitatory synapses that were also immune-positive for Trio. Arrowheads depict puncta of Piccolo or Synaptophysin that were negative for PSD-95 (and thus either inhibitory synapse or transport vesicles) that were also Trio positive. Insets in A-C show higher mag regions of boxed areas. (D) Quantification of Trio puncta that colocalize with PSD-95 positive (+) or negative (-) puncta that were also positive for Piccolo or Synaptophysin puncta. Piccolo or Synaptophysin puncta were separated into PSD-95 positive (+) or negative (-) groups, which were then each scored as positive or negative for Trio. * = p < 0.0005, paired t test comparing PSD-95 positive (+) or PSD-95 negative (-) groups in individual images. n = 10 images for each group.

To verify the synaptic localization of the longer Trio isoforms, we used immuno-fluorescence microscopy of hippocampal neurons grown for 16 days *in vitro* (DIV) to compare the spatial distribution of Trio to those of known synaptic markers. For these experiments, we used the α-C-terminal Trio antibody, as the α-GEF-2 Trio antibody failed to immunostain Trio in cultured neurons (data not shown). As presented in Fig 2A, the α-C-terminal Trio antibody exhibited a punctate pattern that decorated both MAP2-positive dendritic arbors as well as MAP2 negative structures that likely represent axons. The synaptic localization of these Trio positive puncta was explored by triple labeling fixed cultures with antibodies against combinations of Piccolo, Synaptophysin, and PSD-95. Qualitatively, most Trio puncta were observed to co-localize with Piccolo and PSD-95 positive puncta (arrows in Fig 2B). In a quantitative assessment, we found that ~75% of PSD-95-positive Piccolo puncta were Trio positive, while less than 50% of PSD-95-negative Piccolo puncta were Trio positive (Fig 2D, left panel). This later group likely represents Piccolo associated with PTVs in axons or inhibitory synapses [35, 41]. Similar results were obtained when the experiment was performed with antibodies to Synaptophysin and PSD-95 (Fig 2C and 2D), supporting the concept that a significant fraction of the longer Trio isoforms are localized at synapses in mature neurons. In Drosophila, Trio has been shown to regulate synaptic growth in neuromuscular junction (NMJ) [39]. In that study the endogenous protein was not detected by immunofluorescence, but the same antibody detected the transgenically expressed Trio in motor neurons terminals [39] suggesting a presynaptic localization.

**Fig 3:**
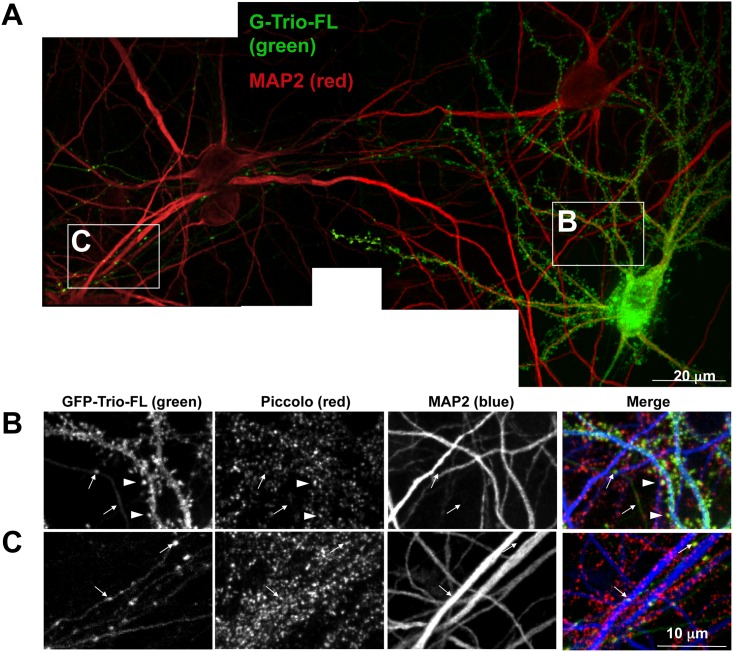
GFP-Trio-FL targets to dendritic spines and presynaptic terminals. (A) Montage of several images of a neuron transfected with GFP-Trio-FL (green) growing near untransfected neurons immunostained with antibodies against the dendritic microtubule associated protein MAP2 (red). (B and C) Higher magnification images from panel A with GFP-Trio-FL (green), Piccolo (red), and MAP2 (blue), illustrating that GFP-Trio-FL targeted to dendritic shafts and spines (arrowheads panel B) as well as to MAP2-negative axons (arrows in panel B). Furthermore, GFP-Trio-FL had a distinctly punctate presynaptic localization in axons as demonstrated by the colocalization of these GFP-Trio-FL puncta (arrows in panel C) with Piccolo positive puncta (red) situated along dendrites of untransfected neurons (C).

As our immuno-fluorescence images could not distinguish between presynaptic or postsynaptic Trio, we transfected cultured hippocampal neurons, at low efficiency, with a Green Fluorescent Protein (GFP)-tagged Trio-FL. As shown in Fig 3A, this protocol allows a single transfected cell to be unambiguously identified in the green channel against a backdrop of untransfected neighbors, as marked by the neuronal- and dendrite-specific marker MAP2 [71]. In this analysis, GFP-Trio-FL exhibited a punctate pattern in the cell soma and dendrites, yet appeared excluded from the nucleus (Fig 3A). At higher magnification, GFP-Trio-FL clearly localized to dendritic shafts and spines (arrowhead in panel B)(Fig 3A and 3B). It was also present in axons (Fig 3A and 3C), identified as thin, long, MAP2 negative processes extending hundreds of microns away from the cell body (arrows in Fig 3B). In the distal axon, GFP-Trio-FL was found in distinct puncta that frequently co-localized with Piccolo adjacent to the MAP2-labeled dendrites of untransfected neurons (arrows in Fig 3C). This analysis demonstrated that Trio, when expressed exogenously in mature cultured neurons, not only localized within dendritic spines, but also localized within presynaptic terminals. Although interesting, the role of Trio in spines was not be explored further in this study.

### The Spectrin repeats of Trio associate with the PBH9-10 regions of Bassoon and Piccolo

**Fig 4:**
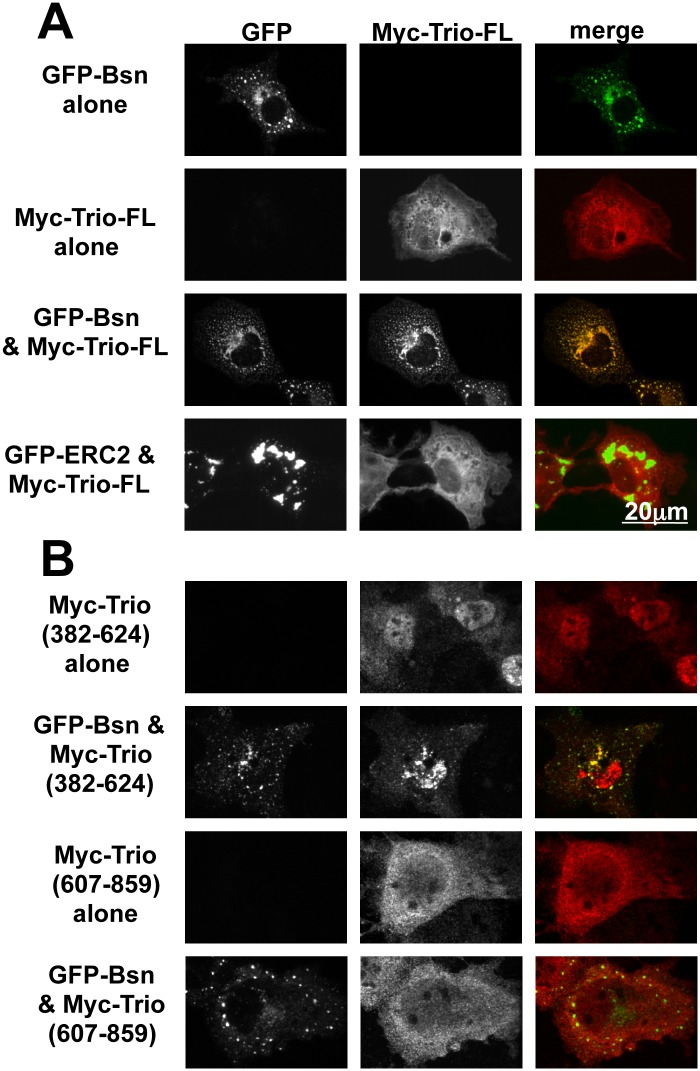
Spectrin repeats 3 and 4 of Trio mediate binding to Bassoon. (A) Examples of COS7 cells co-transfected with full-length (FL) Myc-tagged Trio-FL (red) and GFP tagged Bassoon (GFP-Bsn) or GFP-ELKS2 (green), used to evaluate binding interactions between these proteins. Note that GFP-Bsn effectively clustered Myc-Trio-FL. (B) COS7 cells co-transfected with Myc-tagged Trio polypeptides (red) and GFP-Bsn (green), used to evaluate which domain of Trio mediate binding to Bassoon. Note that Myc-Trio (382–624) clustered with GFP-Bsn, while Myc-Trio (607–859) did not.

To explore whether Bassoon and/or Piccolo direct the presynaptic localization of Trio-FL, we examined whether Myc-tagged Trio-FL could physically associate with a nearly full-length GFP-tagged Bassoon construct [GFP-Bassoon95-3938] [43] in COS7 cells. This was initially accomplished utilizing a clustering assay based on the intrinsic ability of GFP-Bassoon to self-associate and form distinct punctate clusters throughout the cell body of COS7 cells (Fig 4A, top row) as previously described [16, 72, 73]. When expressed alone, Myc-Trio-FL was distributed throughout the cell body, decorating the cell periphery in a striated pattern (Fig 4A, second row). Quite strikingly, when the two proteins were co-expressed, GFP-Bassoon retained its punctual distribution, and Myc-Trio-FL was recruited to these puncta, strongly colocalizing with GFP-Bassoon (Fig 4A, third row). GFP-ELKS2, another CAZ protein that also clusters in COS7 cells [72], failed to recruit Myc-Trio-FL into the GFP-ELKS2 clusters (Fig 4A, bottom row) indicating specificity.

**Fig 5:**
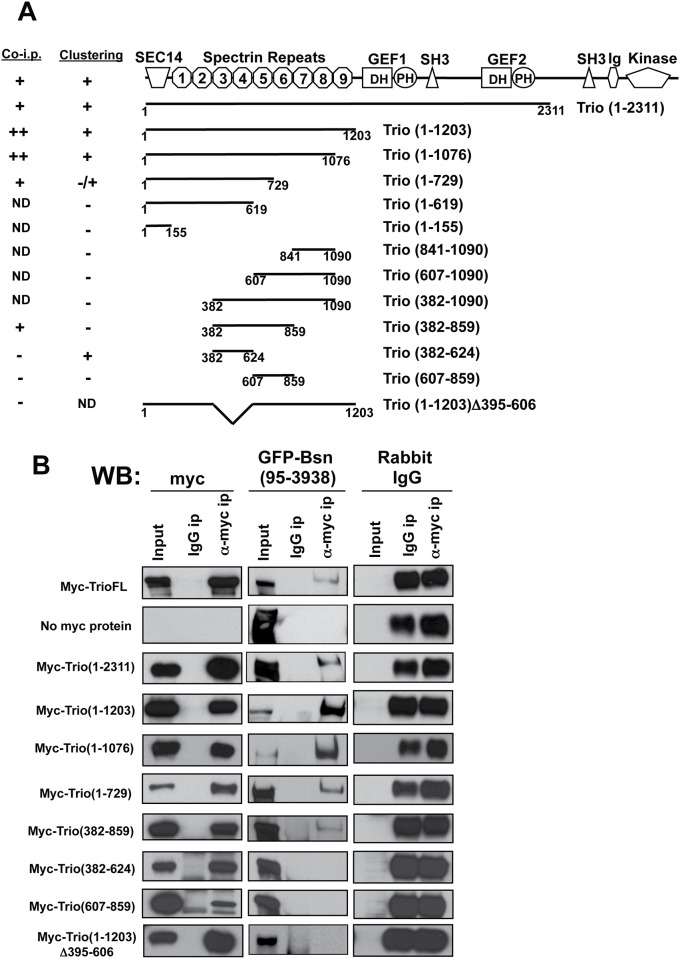
Spectrin repeats 3 and 4 of Trio are required for co-immunoprecipitation (co-IP) with Bassoon (Bsn). (A) Schematic diagram depicting FL-Trio and the polypeptides used in the clustering and co-IP assays. Column labeled “Clustering” summarizes the ability of different Trio mutants to bind GFP-Bsn (95–3938). Column labeled “Co-IP” contains a similar analysis for Trio constructs co-immunoprecipitated with GFP-Bsn (95–3938)(as shown in panel B). ND = not determined. (B) Western blot showing co-IP of GFP-Bsn95-3938 with Myc-Trio polypeptides (using Myc antibody) from COS7 cell lysates. Blots in left column (Myc) show levels of Myc-Trio constructs in total lysates (input), after control IP with rabbit IgG (IgG IP), and after IP with Myc antibody (α-Myc ip). Middle column (GFP-Bsn) shows GFP-Bsn 95–3938 levels for the same conditions, as detected with anti-GFP antibody. Right column shows Rabbit IgG, demonstrating that similar levels of antibody were used in the α-Myc and control conditions.

To define the region of Trio responsible for its binding to Bassoon, we screened a series of Myc-tagged Trio deletion constructs in this clustering assay (Fig 4B). As summarized in Fig 5A, the smallest Trio piece capable of co-clustering with Bassoon (Myc-Trio382-624) contains Spectrin repeats 3 and 4. As with the longer Trio constructs containing this region, Myc-Trio382-624 co-clustered with GFP-Bassoon in COS cells, while the segments lacking repeats 3–4 did not (Fig 4B). While this analysis highlights the importance of Spectrin repeats 3 and 4 in binding Bassoon, we also noted that larger pieces of Trio containing these repeats did not necessarily cluster with Bassoon (Fig 4B). These data suggest that complex intramolecular interactions in Trio may influence its association with Bassoon.

As an alternative strategy to map the region in Trio capable of binding Bassoon, we performed a series of IPs with GFP-Bassoon95-3938 and different segments of Trio co-expressed in COS7 cells. Here, anti-Myc antibodies were used to IP Myc-Trio and the presence of GFP-Bassoon assessed by probing Western blots with antibodies against GFP (Fig 5B). In this assay, GFP-Bassoon was co-immunoprecipitated with Myc-Trio-FL (Fig 5B). Importantly, GFP-Bassoon was neither detected when a non-specific rabbit IgG was used in the IP (Fig 5B, top row), nor when GFP-Bassoon was expressed alone and then precipitated by the anti-Myc antibody (Fig 5B, second row). As in the co-clustering assay, Myc-tagged Trio polypeptides that contain the Spectrin repeat 3 and 4 regions were found to precipitate GFP-Bassoon (results summarized in Fig 5A). For example, Myc-Trio1-729 and Myc-Trio382-859 both precipitated GFP-Bassoon (Fig 5A); this result was particularly interesting because these two proteins clustered poorly with GFP-Bassoon (Fig 5A). Moreover, the minimal region of Trio that clustered with Bassoon (Myc-Trio382-624; Figs 4B and 5A) did not associate with GFP-Bassoon in the IP experiment (Fig 5B). Nonetheless, Myc-Trio1-1203 (Δ395–606), which lacks the minimal clustering region, was incapable of binding to GFP-Bassoon95-3938 (Fig 5B bottom row; compare with Myc-Trio1-1203 on the forth row from top). Together, these data highlight Spectrin repeats 3 and 4 (amino acids 382–624) as a critical region mediating the association of Trio with Bassoon. Our results further suggest that additional regions of Trio both positively and negatively regulate an association with Bassoon.

**Fig 6:**
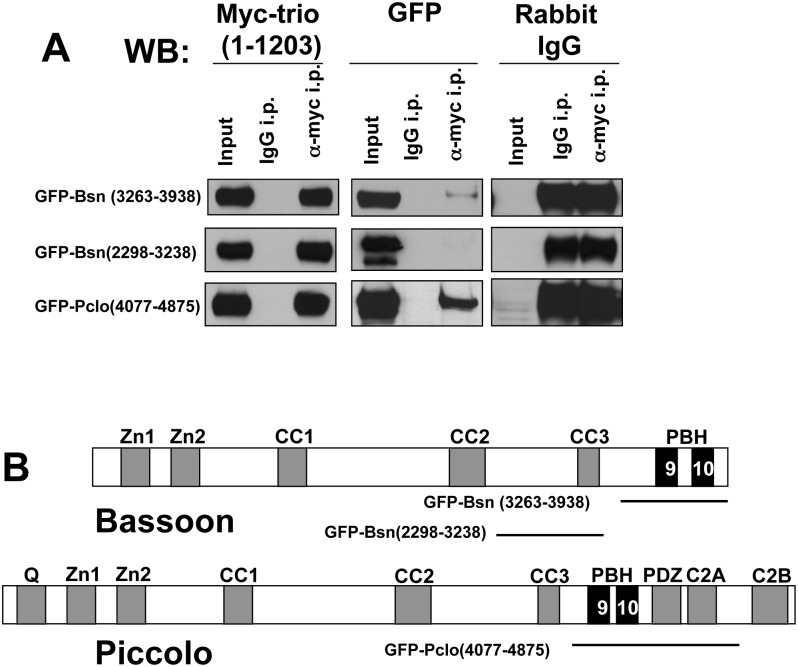
Trio interacts with the PBH9/10 regions in Piccolo and Bassoon. (A) Western blots evaluating co-IP of GFP-Bassoon (Bsn) and Piccolo (Pclo) polypeptides with Myc-Trio1-1203 (using Myc antibody) from COS7 cell lysates. IP and Westerns were performed as in Fig 5B. (B) Schematic of Bassoon (top) and Piccolo (bottom) illustrating the following domains: Zinc finger (Zn), coiled-coil (CC), Piccolo/Bassoon homology (PHB; only 2 of the 10 PHB domains depicted), poly-Q (Q) PDZ, and C2 domains. Below the domain diagram are single lines depicting the regions of Bassoon and Piccolo that bound to Trio in panel A. The PBH 9 and PBH 10 domains are the only conserved domains in this region of these proteins.

In a third set of molecular mapping experiments, we sought to define the region in Bassoon that binds the Spectrin repeat region of Trio. Here, GFP-tagged segments of Bassoon were co-transfected with the 1–1203 region of Trio (Myc-Trio1-1203). After immunoprecipitation with an anti-Myc antibody, anti-GFP Western blotting was used to determine potential co-IP (Fig 6A). This analysis revealed that the C-terminal region of Bassoon (GFP-Bassoon3263-3938) (Fig 6B) bound to Trio (Fig 6A), while GFP-Bassoon2298-3238 (Fig 6A) and other Bassoon polypeptides lacking amino acids 3263–3938 did not (data not shown). As this region of Bassoon contains the Piccolo/Bassoon Homology (PBH) domains 9 and 10 (Fig 6C), we also examined whether the homologous region of Piccolo bound Trio. We observed that the C-terminal PBH9/10 region of Piccolo (amino acids 4077–4875) did in fact co-precipitate with Myc-Trio1-1203 from COS7 cell lysates (Fig 6A) consistent with this region mediating an association with Trio.

### Trio requires its Spectrin 3–4 region for its targeting and tight association with the CAZ

**Fig 7:**
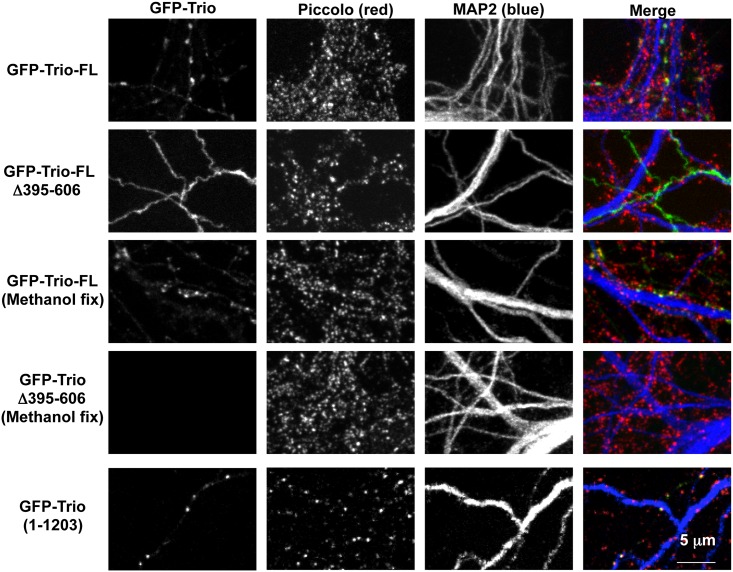
Trio requires Spectrin repeats 3 and 4 to be targeted to presynaptic boutons. Images of axons from neurons transfected with GFP-Trio-FL, GFP-Trio-FL(Δ395–606) or GFP-Trio(1–1203) growing along the dendrites of untransfected cells that have been fixed and immunostained with Piccolo (red) and MAP2 (blue). Neurons were fixed with methanol where indicated or formaldehyde (when fixative is not specified). Note that when GFP-Trio-FL is lacking amino acids 395–606 (Spectrin repeats 3 and 4), it did not localize to the presynapse and was readily extracted by methanol fixation. GFP-Trio (1–1203), which contains all the spectrin repeats, was targeted effectively to presynaptic sites.

The ability of Trio to bind both Bassoon and Piccolo via its spectrin repeats suggests that it may utilize this region to target to the CAZ. To test this hypothesis, we expressed recombinant Trio lacking Spectrin repeats 3 and 4 (GFP-Trio1-1203 (Δ395–606) in hippocampal neurons cultured for 16DIV (Fig 7). Immunofluorescence was then used to assess its ability to localize within presynaptic terminals formed along MAP2-labeled dendrites that were also immune-positive for Piccolo. Here, we found that while GFP-Trio-FL nicely localized within presynaptic boutons (Fig 7, top row; same analysis as Fig 3C), GFP-Trio1-1203 (Δ395–606) remained diffuse throughout the axon (Fig 7, second row).

To assess the ability of recombinant segments of Trio to become stably associated with the CAZ, transfected neurons were fixed in methanol, a condition that retains cytoskeletal proteins (including Piccolo and Bassoon [43]), but extracts soluble cytoplasmic proteins. Importantly, while GFP-Trio-FL was retained within presynaptic boutons following methanol fixation, GFP-Trio1-1203 (Δ395–606) was completely extracted (Fig 7). These data indicate that the stable association of Trio with the presynaptic cytoskeletal matrix required Spectrin repeats 3–4.

As a further measure to test whether the Spectrin repeat region in Trio indeed mediates its presynaptic localization, we immunostained neurons expressing the N-terminal half of Trio (GFP-Trio1-1203) (Fig 5A) with antibodies to Piccolo and MAP2 (Fig 7). As with full-length Trio, within axons, this segment of Trio became selectively localized to presynaptic terminals (Fig 7). Taken together, these data demonstrate that Spectrin repeats 3 and 4 of Trio are required for presynaptic localization of Trio and imply that Trio requires its association with Bassoon/Piccolo to localize to presynaptic terminals.

### The C-terminal half of Trio negatively regulates its association with Piccolo and Bassoon

**Fig 8:**
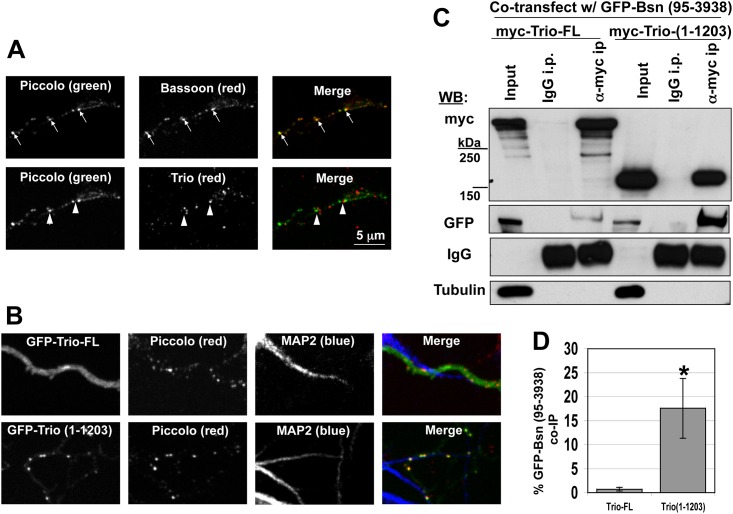
The C-terminus of Trio inhibits binding with Piccolo/Bassoon on PTVs. (A) Images of axons from immature neurons (5DIV) immunostained with Piccolo, Bassoon, and Trio. In these neurons, Piccolo and Bassoon co-localized (arrows in top panel), while Piccolo and Trio did not (arrowheads in bottom panel). (B) Axons from neurons (5DIV) expressing GFP-Trio-FL or GFP-Trio (1–1203), stained for Piccolo and MAP2. In these immature axons, GFP-Trio-FL remained diffuse, while GFP-Trio (1–1203) was punctate and colocalized with Piccolo. (C) Western blot of co-immunoprecipitation (IP) experiments comparing the ability of Myc-Trio-FL and Myc-Trio (1–1203) to co-IP with GFP-tagged Bassoon [GFP-Bsn(95–3938)]. Tubulin was included as an additional control demonstrating a lack of non-specific co-IP. (D) Quantification of panel C, obtained by dividing the pixel intensity of the GFP-Bassoon band in the anti-Myc lane by the GFP-Bsn band in the input lane and normalizing the results to the total amount of GFP-Bsn present in the lysate. Trio1-1203 immunoprecipitated Bassoon more effectively than full-length (FL) Trio. * = p < 0.05. n = 5.

The ability of Trio to bind Bassoon and Piccolo within presynaptic active zones suggests that it may also utilize these interactions for delivery to nascent and/or mature synapses. To test this hypothesis, we first compared the spatial distribution of endogenous Trio with Piccolo and Bassoon in immature neurons (5DIV), a developmental stage preceding synaptogenesis in which PTVs are plentiful [35]. In hippocampal neurons at this stage, Bassoon and Piccolo were highly colocalized along extending axons, exhibiting a punctate pattern consistent with their association with PTVs (Fig 8A, top row). The long Trio isoforms (recognized by the α-C-term Trio antibody) also exhibited a punctate pattern in these axons; however, Trio puncta did not co-localize with Piccolo (Fig 8A, bottom row). Furthermore, in contrast to mature neurons (Fig 3), GFP-Trio-FL expressed in immature neurons did not co-localize with Piccolo-positive puncta, rather it was mostly diffuse throughout axons (Fig 8B, top) and the rest of the neuron (data not shown). Conversely, GFP-Trio (1–1203), which contains the N-terminal Spectrin repeats of Trio and the presynaptic targeting domain (Fig 7), co-localized with Piccolo puncta in immature neurons (Fig 8B, bottom panel). These data suggest that this region of Trio acquires the capacity to bind Bassoon and/or Piccolo on PTVs, and that the C-terminal segment of Trio negatively regulate this interaction.

To directly test the hypothesis, we quantitatively compared the capacity of GFP-Bassoon to co-immunoprecipitate with Myc-Trio-FL vs. Myc-Trio (1–1203) (Fig 8C and 8D). In these experiments, 10–20 times more of the available GFP-Bassoon was co-immuno-precipitated by Myc-Trio (1–1203) compared to full-length Myc-Trio-FL (Fig 8D). These data suggest that the N-terminal Trio (1–1203) construct exists in a conformational state that readily allows a tighter association with Bassoon/Piccolo than full-length Trio, and supports the concept that the C-terminal half of Trio serves as a negative regulator of its Bassoon/Piccolo interaction before arriving at nascent synapses.

## Discussion

### The Rho-GEF Trio is a novel interacting partner of Bassoon and Piccolo

Piccolo and Bassoon are structurally related scaffold proteins [44] that localize precisely to the cytomatrix at the active zone (CAZ) of synapses [66, 68]. There has been a great deal of interest in the function of these proteins, as they are among the first to arrive at nascent synapses [35, 67] and exhibit features of structural proteins that may regulate both the assembly of the AZ as well as the release of neurotransmitter [2, 74]. Loss-of-function studies in mice demonstrated that Bassoon has a critical role in the attachment of the ribbon to the presynaptic membrane and arciform density in photoreceptor cells [9, 19]. While its role as a structural protein at conventional synapses is less obvious, it is critically involved in regulation synaptic transmission through the localization of voltage gated calcium channels [14, 33]. Loss-of-function studies with Piccolo, in which its expression at hippocampal synapses was eliminated using interference RNAs, resulted in an enhanced activity-dependent dispersion of Synapsin1a and enhanced rate of SV exocytosis, implicating Piccolo as a negative regulator of neurotransmitter release [12]. These changes in presynaptic function can be attributed to the reduced activity dependent assembly of presynaptic F-Actin [30], a cytoskeletal protein critical for regulating SV release probability, SV endocytosis, and SV delivery from the reserve pool to the readily releasable pool [25]. Intriguingly, several Piccolo binding partners, including Abp1, GIT1, Profilin, and Daam1, are involved in the regulated assembly of F-actin [20–22]. These data suggest that one function of Piccolo within presynaptic boutons is to scaffold molecules involved in the regulated assembly of actin. This present study further supports this concept by demonstrating that Piccolo and Bassoon direct the Rho-GEF Trio, a known regulator of actin [53, 60], to presynaptic boutons.

Our work provides several lines of evidence that Trio has a physical interaction with Bassoon and Piccolo, and that this interaction is regulated. First, we find that Trio co-fractionated with Piccolo and Bassoon in a three-step protocol that separates proteins/membrane based on buoyant density, size, and charge (S1–S3 Figs). Second, using MudPIT, we were able to detect the presence of Trio in a Piccolo/Bassoon complex. Third, exogenously expressed Trio and Bassoon were found to form complexes in heterologous cells, as detected by both immunoprecipitation and immunofluorescence. Finally, as discussed below, we find that truncated forms of Trio (particularly the first 1203 amino acids) appear to have a higher binding affinity than full length Trio for Piccolo/Bassoon.

### Trio colocalizes with Piccolo and Bassoon in mature synapses

An important question raised by our cellular and biochemical studies is when during neuronal development Trio associates with Piccolo and/or Bassoon. Our previous studies on Piccolo and Bassoon demonstrate that these active zone proteins become associated with membranes within the Golgi [65], where they are packaged together in a specialized 80 nm dense core PTV [35], allowing them to be delivered to nascent synapses in a quantal manner [52]. Spatially, these transport vesicles accumulate in axons and growth cones, and are eventually incorporated into synapses [35, 41, 52]. Trio is also found in immature axons [69], and appears to be enriched in the growth cones of extending PC12 cell neurites [54] and in growth cones of rat hippocampal neurons [75]. In addition, Trio was found to promote the fusion of large dense core vesicles in PC12 cells through its association with PAM, a secretory granule membrane protein [76]. While we envision an analogous role for Trio in promoting the fusion of PTVs during nascent synapse formation in hippocampal neurons, Trio does not appear to be co-transported with Piccolo and Bassoon on PTVs (Fig 8), and thus may not play a role in the fusion of these precursor vesicles with the plasma membrane. Intriguingly, we observed a striking colocalization of Trio with Piccolo and Bassoon within presynaptic boutons (Figs 2 and 3), suggesting that Trio may have a more relevant role in mature synapses, perhaps by regulating the dynamic assembly of presynaptic F-actin.

A synaptic localization for Trio is directly supported by studies demonstrating the presence of Trio in purified SJ/PSD preparations, dendritic spines, and presynaptic boutons (Figs 1–3). Intriguingly, by employing GFP-tagged versions of Trio, we were able to demonstrate that Trio, present in presynaptic boutons (Fig 3), becomes resistant to methanol extraction (Fig 7), consistent with its association with the AZ cytoskeletal matrix within these boutons, similar to Piccolo and Bassoon [43]. Moreover, we were able to show that Trio’s presynaptic localization and tight association with the cytomatrix requires the Spectrin repeats 3 and 4.

### Trio interaction with Piccolo and Bassoon is regulated

The observation of a strong colocalization of Trio with Bassoon and Piccolo in synapses of mature but not immature neurons raises the possibility that Trio can exist in a conformational state that limits its association with Piccolo/Bassoon within neurons. Initial clues supporting this concept came from analyzing the expression of either full length or the N-terminal half of Trio (1–1203) in young neurons. Here, we found a precise colocalization between GFP-Trio (1–1203) and PTVs (Fig 8B), while little overlap was detected with GFP-Trio-FL (Fig 8A and 8B). These data imply that the C-terminal PBH9/10 region is available on Piccolo and Bassoon during transport, and that the absence of FL-Trio on PTVs may be related to the conformational state of Trio, e.g. that its C-terminal half masks the Piccolo/Bassoon binding site within the Spectrin 3–4 repeats. Consistent with this model, we found that Myc-Trio (1–1203) more efficiently (>10 fold) co-immunoprecipitated with GFP-Bassoon than full length Trio (Myc-Trio-FL) (Fig 8C and 8D).

Taken together, these data suggest that the interactions among Trio, Piccolo, and Bassoon are tightly regulated and only occur within specific subcompartments in neurons, e.g. presynaptic boutons. In this model, Piccolo and Bassoon use their multidomain structure to become core components of the pre-synaptic active zone. Trio, on the other hand, is clearly a more dynamic multi-functional protein, with roles in a variety of neuronal compartments, including growth cones, dendritic spines, and the presynaptic AZ. For example, *Drosophila* studies have demonstrated that Trio is necessary for the effect of retrograde signals at the neuromuscular junction acting on the motor neuron [39], and recent studies in mammalian systems have demonstrated that Trio has roles in regulating AMPA-type glutamate receptors postsynaptically [77]. Therefore, we would expect that, at any one moment in a neuron, only a small percentage of the available Trio would be bound to Piccolo or Bassoon. This conclusion is consistent with our inability to detect Trio by Western blot in Piccolo IPs. However, using the more sensitive MudPIT technique [45, 78], we did succeed in detecting the small percentage of cellular Trio that interacts with Piccolo and Bassoon under steady-state conditions.

### Potential role of Trio at presynaptic active zones

The ability of presynaptic active zone proteins to tether Trio isoforms at neurotransmitter release sites suggests that Trio might be involved in aspects of presynaptic function. The best characterized facets of Trio function relate to its two GEF domains [59], implicated in the activation of Rho family GTPases [53] including RhoA, RhoG, Rac, and cdc42. Specifically, the GEF1 domain in Trio has been found to directly activate RhoG [42] and indirectly activate cdc42 [79] and Rac [42], while its GEF2 domain activates RhoA [54]. These are all part of signaling pathways known to regulate the assembly of F-actin in a spatially restricted manner to control morphogenesis, polarity, and motility of cells [60]. Studies in *Drosophila* and *C*. *elegans* demonstrate that Trio coordinates these various F-actin regulatory signals to control complex physiological processes such as axonal pathfinding and target recognition [53, 57, 80–84].

At present, the functional significance of Trio’s localization within the presynaptic active zone is unclear. Several studies reveal that a complex of Trio with LAR, a protein tyrosine phosphatase, modifies axon guidance [42, 80]. More recently, a cascade of molecules including Trio, LAR, and diaphanous (a formin) were found to regulate the growth of presynaptic boutons at the *Drosophila* neuromuscular junction by modulating the assembly of both microtubules and microfilaments [40]. These data indicate that axonally targeted Trio might participate in functions beyond the guidance of axons, and may influence the regulated assembly of actin in presynaptic boutons through interactions with Piccolo and Bassoon. In fact nascent synapse formation is actin dependent [85]. Intriguingly, we recently showed that the formin ‘Daam1’, a non-canonical Wnt signaling component, interacts with Piccolo and is involved in the activity dependent assembly of F-actin [2, 23]. It is very well known that Wnt signaling participates in various facets of neuronal development, including neuronal polarity, migration, guidance, dendrite development, and synaptogenesis [86, 87]. In cerebellar granule cells neurons Wnt-7a increases the levels of Synapsin-I at synapses [88], and both Wnt-7a and Wnt-3a increase clustering of Bassoon in young hippocampal neurons after 1 h incubation involving a signaling mechanism upstream of GSK-β [89, 90]. Therefore, Wnt signaling might regulate the interaction of Piccolo, Bassoon, Daam1, and Trio both in young neurons, modulating presynaptic assembly and thereafter, in mature neurons, regulating synaptic plasticity. Finally, a recent paper describing Trio’s role in controlling post-synaptic trafficking events underlying long-term potentiation (LTP) clearly showed redundancy between Trio and the highly related protein Kalirin [77]. Thus, future functional studies on Trio in mammalian systems will need to take into account potential redundancy between Trio and Kalirin.

In summary, our data reveal that the Rho-GEF Trio is a novel component of presynaptic AZ and utilizes its Spectrin repeats to direct its association with structural elements within the cytoskeletal matrix assembled at neurotransmitter release sites. Intriguingly, these interactions place Trio in a unique environment that will allow it to regulate the dynamic assembly of presynaptic F-actin in response to changes in signaling cascades that either directly regulate the release of neurotransmitter and/or the growth/maturation of presynaptic boutons.

## Supporting Information

S1 Table List of rat (*Rattus norvegicus*) proteins identified by MudPIT analysis. 24 proteins were both identified in the Piccolo immunoprecipitation (IP) and were absent in the control IgG (suggesting specific Piccolo interaction). An additional 398 proteins were identified in the column fractionation experiment but were not identified in any IP experiment. For completeness, additional categories of likely non-specific proteins are included in the table. Highlighted in orange are the three proteins selectively enriched in both the Piccolo IP and the 290 mM fraction. Descriptions of abbreviations are found at the top of the table.
(XLSX)

S1 Fig Identification of Trio as a protein that co-fractionates with Piccolo and Bassoon. (A) This schematic depicts the workflow used to generate a fraction enriched in Piccolo and Bassoon starting with day 4 (P4) rat brain lysates. (B) The final step of the purification described in Panel A, when material was passed over an anion exchange Q column, required some level of optimization. Here, the fraction from step 1 in panel A (sucrose density centrifugation) was added to the Q column and washed with an excess of 300 mM sucrose. Then a linear gradient from 0–600 mM NaCl was added to successively elute proteins, and a final 1M NaCl elution was used to strip the column of material bound by hydrophilic association. Fractions were run on SDS-PAGE and Western blotted for the protein depicted to the left of the gels. Of note, the three proteins monitored, ELKS2, Bassoon (Bsn), and Synaptophysin (Syph) had 3 different elution profiles; this result was exploited for further optimization and purification (see S2 and S3 Figs).
(EPS)

S2 Fig Optimization of Q column leads to separation of CAZ and SV proteins. During these optimization experiments, material between the 0.3M and 0.8M interface of a sucrose density gradient centrifugation (step #1 in S1A Fig) was passed over a Q column. Stepwise elutions with indicated NaCl concentrations were used. (A) Total amount of protein in each of the fractions collected as measured by the Bio-Rad Protein Assay reagent. (B) Fractions were subjected to SDS-PAGE and Western blotted for proteins indicated to the left of the figure. These experiments yielded an ELKS2-positive fraction devoid of other markers (210 mM); a Piccolo (Pclo) and Bassoon (Bsn) fraction, which also contained ELKS2 (290 mM); and a Synaptophysin (Syph)-containing SV protein fraction (350 mM).
(EPS)

S3 Fig Western blots of Q-column fractions and IPs of postnatal day 2 brain light membrane fractions. (A) After optimization, the final step of the three-step protocol from panel A was stepwise increases in NaCl (See S2B Fig) to elute pools of protein. These fractions were then run on SDS-PAGE and Western blotted for the protein depicted to the left of the gels. The 290 mM NaCl fraction was enriched in Piccolo (Pclo) and Bassoon (Bsn) and contains ELKS2, but was devoid of the synaptic vesicle (SV) proteins Synaptophysin (Syph) and Synaptotagmin (Syn-tag). Trio, detected using the α-GEF2 Ab, was enriched in the Pclo/Bsn-positive 290 mM NaCl fraction. (B) Western blots of immunoprecipitations with either rabbit IgG or rabbit Piccolo (Pclo) antibodies, from light membrane fractions prepared by sucrose centrifugation from postnatal day 2 rat forebrain, probed with antibodies to Piccolo (Pclo), Bassoon (Bsn), ELKS2, RIM1/2 or Synaptophysin (Syphy). Data demonstrate that the 290 mM NaCl fraction analyzed by MudPIT contained active zone proteins but not synaptic vesicle proteins.
(EPS)
